# Anticipated and experienced discrimination amongst people with schizophrenia, bipolar disorder and major depressive disorder: a cross sectional study

**DOI:** 10.1186/1471-244X-14-157

**Published:** 2014-05-29

**Authors:** Simone Farrelly, Sarah Clement, Jheanell Gabbidon, Debra Jeffery, Lisa Dockery, Francesca Lassman, Elaine Brohan, R Claire Henderson, Paul Williams, Louise M Howard, Graham Thornicroft

**Affiliations:** 1 Section of Community Mental Health, Health Service and Population Research Department, King’s College London, Institute of Psychiatry, De Crespigney Park, Box PO29, SE5 8AF London, UK; 2 Adelphi Values, Adelphi Mill, Grimshaw Lane, SK10 5JB Bollington, Cheshire, UK

**Keywords:** Schizophrenia, Depression, Bipolar, Discrimination, Gender, Ethnicity, Stigma

## Abstract

**Background:**

The unfair treatment of individuals with severe mental illness has been linked to poorer physical and mental health outcomes. Additionally, anticipation of discrimination may lead some individuals to avoid participation in particular life areas, leading to greater isolation and social marginalisation. This study aimed to establish the levels and clinical and socio-demographic associations of anticipated and experienced discrimination amongst those diagnosed with a schizophrenia and comparator severe mental illnesses (bipolar and major depressive disorders).

**Methods:**

This study was a cross-sectional analysis of anticipated and experienced discrimination from 202 individuals in South London (47% with schizophrenia, 32% with depression and 20% with bipolar disorder).

**Results:**

93% of the sample anticipated discrimination and 87% of participants had experienced discrimination in at least one area of life in the previous year. There was a significant association between the anticipation and the experience of discrimination. Higher levels of experienced discrimination were reported by those of a mixed ethnicity, and those with higher levels of education. Women anticipated more discrimination than men. Neither diagnosis nor levels of functioning were associated with the extent of discrimination. Clinical symptoms of anxiety, depression and suspiciousness were associated with more experienced and anticipated discrimination respectively.

**Conclusions:**

The unfair treatment of individuals with severe mental illnesses remains unacceptably common. Population level interventions are needed to reduce levels of discrimination and to safeguard individuals. Interventions are also required to assist those with severe mental illness to reduce internalised stigma and social avoidance.

## Background

Mental and behavioural disorders account for almost a quarter of the non-fatal health- related disability worldwide [1] at a cost of approximately £11.8 billion per year in England [2]. A proportion of this cost derives from missed education and employment opportunities, homelessness, or poor physical health leading to reduced life expectancy (which for severe mental illness (SMI) is 15–20 years less than the general population) [2]. One potential reason for such poor outcomes is ‘experienced discrimination’ , which can be defined as reported unfair treatment due to having a diagnosis of a mental illness [3,4]. Further, the anticipation of discrimination may lead to avoidance of important life areas (such as employment, education or healthcare).

Data from three studies suggest high rates of mental illness-related discrimination are common. In 2009, the INDIGO schizophrenia study [4] reported the lifetime experiences of discrimination amongst 732 individuals diagnosed with schizophrenia in 27 countries. High rates of discrimination were consistent across countries. 47% of participants had experienced discrimination in making or keeping friends, 43% from family members and 29% in finding/keeping a job. The same group examined experienced discrimination amongst 1087 individuals diagnosed with major depressive disorder in 35 countries [5] and found 79% of participants experienced discrimination in at least one area of life. Finally, the Viewpoint annual cross-sectional survey of users of secondary mental health services in England [6] suggests that while rates of discrimination may be improving, 88% of those surveyed in 2011 had experienced discrimination in at least one area of life in the previous 12 months [7].

These studies also captured the extent to which the anticipation of discrimination stopped people from participating in important areas of life, including work, education and personal relationships. Interestingly, while there was an association between the anticipation of discrimination and prior experience of discrimination, that was not true in all cases. For example, in the INDIGO schizophrenia study one third of participants anticipated discrimination in work despite having had no prior experience of discrimination in this area [4,8].

The impact of experienced and anticipated discrimination can be profound. For example, in a qualitative analysis of the INDIGO schizophrenia study [9], many participants described feeling ‘shunned’ and ‘mocked’ by their communities, resulting in or exacerbating social withdrawal. Further, two meta-analyses indicate that experienced discrimination (due to any social attribute) is linked with poorer mental and physical health [10,11].

These studies have been seminal in understanding the extent of experienced discrimination internationally, however, with relatively low sample sizes in the international studies [4,5] and low response rates [7], the generalisability of these findings is unclear. Further, anticipated discrimination was measured by four items which captured a behavioural consequence of discrimination i.e., avoidance; measurement of actual anticipation of discrimination using a validated measure is required. In addition, little is known about the predictors of discrimination, e.g., are people with schizophrenia more likely to experience discrimination than those with a diagnosis of bipolar disorder? Are men or women more likely to anticipate discrimination? Finally, questions remain regarding the influence of functioning and psychopathology on discrimination [8,12]. For example, do people with more severe symptoms experience more discrimination than those less impaired? Are feelings of hopelessness associated with increased reporting of discrimination?

This paper presents the results of a cross-sectional study investigating the patterns and associations of experienced and anticipated discrimination for people with SMI and addresses four research questions:

1. What is the nature and severity of experienced and anticipated discrimination reported by people with schizophrenia, bipolar disorder and major depressive disorder?

2. What are the associations between experienced and anticipated discrimination?

3. What are the socio-demographic predictors of experienced and anticipated discrimination - in particular are there different rates of discrimination according to ethnicity, diagnosis, age, gender, or education?

4. Are symptoms, functioning or hopelessness associated with rates of experienced or anticipated discrimination?

## Methods

The MIRIAD (Mental Illness-Related Investigations on Discrimination) study was a cross-sectional study of 200 individuals using secondary mental health services in South London. Data were collected between September 2011 and October 2012. The study was approved by the East of England/Essex 2 Research Ethics Committee (ref 11/EE/0052).

### Recruitment and sample

Inclusion criteria were: aged at least 18 years; a clinical diagnosis of either Major Depression, Bipolar or Schizophrenia spectrum disorders; self-defined Black, White or Mixed (Black and White) ethnicity; current treatment with a community mental health team (CMHT); sufficiently fluent in English to provide informed consent; and sufficiently well for participation to not pose a risk to their or others’ health or safety. We did not include Asian ethnicities due to low prevalence numbers in the target area.

Clinicians were provided with a list of eligible service users and asked if the service user was sufficiently well to participate. A letter was posted to eligible service users inviting them to contact the research team if they were interested in participating. This letter was followed by a reminder flyer if there had been no response within one month.

### Data collection

Research Assistants interviewed participants usually over two sessions (range 1–4). Participants received £15 ($23 USD) per sitting for their time. The interview schedule collected demographic and clinical information and contained a battery of measures on stigma, discrimination and access to physical and mental health care; those relevant to this paper are detailed below. Clinical data were extracted from patient records.

*Discrimination and Stigma Scale (DISC)*[13]: an interviewer-delivered measure of experiences of discrimination (‘unfair treatment’) in the last 12 months due to a diagnosis of a mental illness. Participants report experiences of discrimination across 21 areas including employment, dating or intimate relationships, on a 4-point Likert scale. The DISC has good psychometric properties [13]. A ‘severity’ score (range 0–3) was calculated by adding each item score and dividing by the number of applicable, non-missing items. A count score (range 0–21) was calculated by counting the number of items where the participant reported any degree of discrimination.

*Questionnaire on Anticipated Discrimination (QUAD)*[14]: a self-complete measure comprising 14 items assessing the extent to which participants expect to be treated unfairly in areas of life similar to the DISC. Each item is scored on a 4-point Likert scale ranging from 0 (Strongly disagree) to 3 (Strongly agree). Psychometric analyses indicate good internal consistency and construct validity [14]. A ‘severity’ score (range 0–3) was calculated by adding each item score and dividing by the number of applicable, non-missing items. A count score of the number life areas of anticipated discrimination was calculated.

*The Brief Psychiatric Rating Scale (BPRS;*[15]*)*: comprises 18 items addressing symptomatology. The scale is widely used and is reliable and valid [15]. Three subscales were calculated according to established criteria [15,16]: Anxiety & Depression, Hostility & Suspiciousness, and Thinking & Perception.

*Global Assessment of Functioning (GAF;*[17]*)* captured current functioning rated by the service users’ main professional caregiver. Rated on a scale of 0–100, it is the most frequently used measure of functioning in the mental health field and has good validity and inter-rater reliability [18].

*Beck Hopelessness Scale (BHS;*[19]*)*: is a self-complete measure assessing hopelessness. The version used in this study had a 5-point Likert (ranging from 1 ‘strongly agree’ to 5 ‘strongly disagree’) on 20 items. A total score was calculated by summing the items (possible range 20 to 100).

*Internalised Stigma of Mental Illness Scale (ISMI;*[20]*)*: a 29-item measure measuring service users’ experience of internalised stigma, rated on a 4-point Likert scale. Strong internal consistency and test–retest reliability have been reported [20]. There are five subscales including a five item ‘Discrimination Experience’ subscale, which due to being conceptually similar to the DISC was excluded. A total score was generated by summing the remaining 24 items.

*Multigroup Ethnic Identity Scale (MEIM;*[21]*)*: a 12 item self-report measure of Ethnic identity, two items rated on a 5-point Likert scale and remaining ten on a 4-point Likert Scale. It has good psychometric properties [21].

### Data analysis

Analyses used Stata version 11. Rates of experienced and anticipated discrimination overall and by life area were assessed using frequency analyses. The relationship between experienced and anticipated discrimination was assessed with Spearman’s rho due to the non-normality of data. Associations between demographic characteristics and severity of experienced and anticipated discrimination were investigated using robust multiple regression to account for non-normality of data. These analyses additionally adjusted for the degree of functioning and symptomatology. Variables were omitted from final models if found not to contribute significantly using likelihood ratio tests. After these preliminary analyses, we conducted post-hoc analyses to test emerging hypotheses regarding the findings. We examined rates of experienced discrimination by ethnic identity and internalised stigma using ANOVA tests.

## Results

4233 service users were screened for eligibility. 1345 (31.7%) were eligible and were invited to participate. 207 (15.4%) service users provided written and informed consent. There were no differences between eligible consenting and eligible non-consenting service users in terms of diagnoses, age, gender and ethnicity. Five service users were excluded after interview due to incorrect diagnoses (n = 4) or incomplete data (n = 1), leaving 202 participants. Their socio-demographic and clinical characteristics are shown in Table 1.

**Table 1:** Socio-demographic and clinical characteristics of sample

**Variable**	**Categories**	**Total n = 202**	**Percentage**
Gender	Male	92	45.5%
	Female	110	54.5%
Ethnicity (self-defined)	Black	77	38.1%
	White	108	53.5%
	Mixed	17	8.4%
Age (years)	Mean (sd; range)	202	41.8 (11.1; 19–67)
Employment status	Employed	46	22.8%
	Not employed	126	62.4%
	Student/Training/Volunteer	25	12.4%
	Missing	5	2.5%
Education level	No qualifications	25	12.4%
	Qualifications usually taken at age 16	50	24.8%
	A-levels/Vocational	67	33.2%
	Degree or higher	60	29.7%
Relationship status	Single	128	63.4%
	Married/Partner	45	22.3%
	Divorced/Widowed	29	14.4%
Children	Yes	86	42.5%
	No	116	57.5%
Diagnosis from notes	Bipolar disorder	41	20.3%
	Depression	65	32.2%
	Schizophrenia Spectrum	96	47.5%
Psychiatric hospital admissions	Ever admitted?	139	68.8%
	Admitted in last 12 months	35	17.8%
	Compulsory admission in last 12 months	14	6.9%
Years since first contact with mental health services	Mean (sd; range)	201	15.1 (11.1; 0–46)

*Abbreviations*: *sd* standard deviation.

### Rates of experienced discrimination

**Figure 1:**
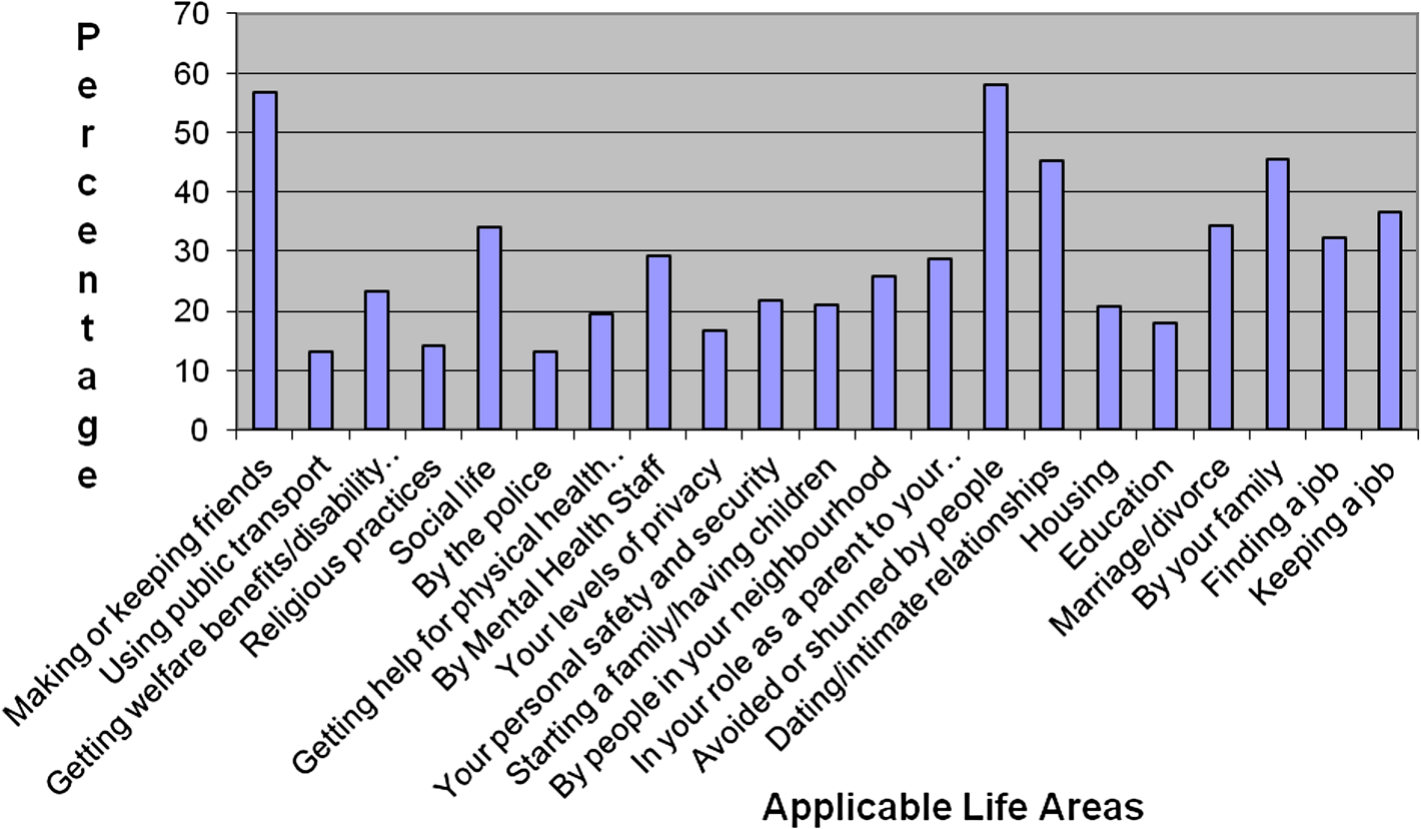
Experienced discrimination by area.

87.6% of the total sample had experienced discrimination in at least one area in the last 12 months. The median number of areas was 5 (range 0–15). Participants indicated if an area was not applicable them, if for example, they had not had the opportunity to partake in that area in the last 12 months. The most frequent areas rated as not applicable were ‘marriage/divorce’ (83%), ‘keeping a job’ (63%), ‘role as a parent’ (58%), ‘finding a job’ (57%) and ‘education’ (56%). The percentage of participants reporting experienced discrimination in each applicable area is shown in Figure 1.

The median severity of experienced discrimination for the total sample was 0.48 (range 0 – 2.28). For those who had reported at least some experienced discrimination the median severity was 0.56 (range 0.06 – 2.28).

### Rates of anticipated discrimination

**Figure 2:**
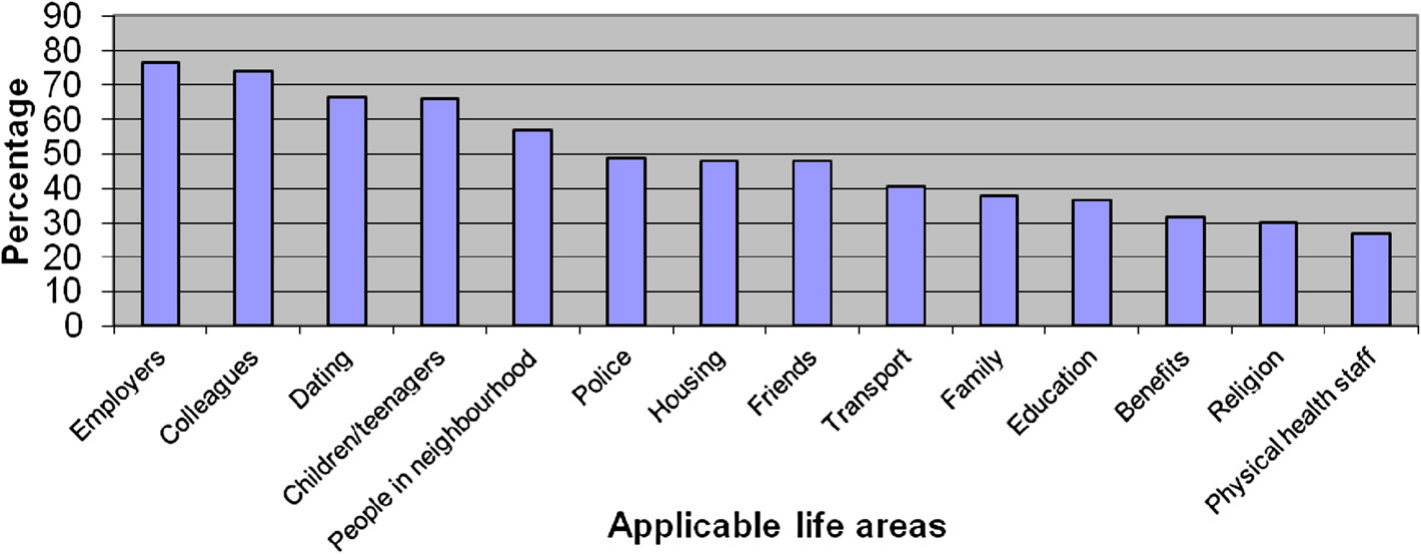
Anticipated discrimination by area.

Almost the entire sample (92.6%) reported anticipated discrimination in at least one area. The median number of areas was 7 (range 0–14). Figure 2 presents the percentage of participants reporting anticipated discrimination in each applicable area. The median severity score for the total sample was 1.50 (range 0 – 2.93) and 1.57 (range 0.36 – 2.93) amongst those who reported at least some anticipated discrimination (n = 186).

### Association between anticipated and experienced discrimination

There was a moderate correlation between: the severity of anticipated and experienced discrimination (n = 199, rho = 0.33 p < 0.001) and the number of areas of anticipated and experienced discrimination (n = 202, rho = 0.42, p < 0.001). We tested the relationship between experienced and anticipated discrimination for related applicable areas. For all areas except ‘Education’ (p = 0.32) and ‘Employment’ (finding a job: p = 0.19; keeping a job: p = 0.19), there was a significant (mostly moderate) association.

### Demographic associations of experienced and anticipated discrimination

Two regression models were fitted with the severity of experienced and anticipated discrimination as dependent variables, and age, gender, ethnicity, and diagnosis as independent variables. These models were adjusted for levels of symptomatology (BPRS subscales), hopelessness (BHS) and functioning (GAF). In both models, hopelessness and functioning did not make a significant contribution and were excluded.

Increased severity of experienced discrimination was associated with higher levels of education, being of Mixed ethnicity (compared to White ethnicity; Black did not differ from Mixed or White), and the Anxiety & Depression subscale of the BPRS (see Table 2). The model accounted for 16.9% of the variance.

**Table 2:** Associations of experienced discrimination

**Severity of experienced discrimination**	**Coefficient**	**Robust standard error**	**t**	**P > t**	**95% confidence interval**	
Females	0.031	0.072	0.44	0.663	−0.109	0.172
Age	0.006	0.003	1.74	0.084	−0.001	0.012
Education – post 16 vs none/up to 16	0.177	0.076	2.30	0.022	0.025	0.328
Ethnicity						
Mixed vs white	0.293	0.139	2.11	0.036	0.019	0.567
Black vs white	0.132	0.083	1.58	0.115	−0.032	0.296
Diagnosis						
Bipolar vs Scz Spec	0.151	0.099	1.51	0.132	−0.045	0.347
MDD vs Scz Spec	−0.005	0.106	−0.05	0.959	−0.215	0.204
Psychopathology						
Hostility & Suspiciousness	0.002	0.018	0.11	0.916	−0.034	0.038
Thinking & Perception	0.012	0.014	0.81	0.418	−0.016	0.038
Anxiety & Depression	0.025	0.007	3.23	0.001	0.009	0.039
_constant	−0.339	0.236	−1.44	0.152	−0.805	0.126

*Abbreviations*: *MDD* Major Depressive Disorder, *Scz Spec* Schizophrenia Spectrum disorder, *vs* versus reference category.

Model: F (10, 187) = 3.55, p = 0.0003.

Specific DISC areas were investigated by ethnicity. The Mixed ethnicity group reported more discrimination in ‘making/keeping friends’ than both the Black and White ethnic groups, and more discrimination in physical and mental health care than the White group. We hypothesised the increased severity in experienced discrimination by the Mixed ethnicity group may partly have been explained by either lower levels of ethnic identity or higher levels of internalised stigma. Exploratory post-hoc analyses supported the latter hypothesis (mean (sd) ISMI total: Mixed: 63.3 (10.4); White 54.8 (10.8); Black 54.8 (11.1). F (2) = 4.45, p = 0.012), but not the former.

Increased levels of anticipated discrimination were associated with being older, female and higher levels of Hostility & Suspiciousness (BPRS) - see Table 3. No other demographic or clinical associations significantly contributed to the model. The model accounted for 17.7% of the variance.

**Table 3:** Associations of anticipated discrimination

**Severity of anticipated discrimination**	**Coefficient**	**Robust standard error**	**t**	**P > t**	**95% confidence interval**	
Females vs Males	.203	.077	2.61	0.010	.049	.356
Age	.007	.004	1.99	0.049	.001	.014
Education – Post 16 vs none/up to 16	-.124	.079	−1.57	0.119	-.280	.032
Ethnicity						
Mixed vs White	.017	.173	0.10	0.920	-.324	.358
Black vs white	.115	.097	1.18	0.238	-.076	.306
Diagnosis						
Bipolar vs Scz Spec	-.062	.109	−0.57	0.572	-.278	.154
MDD vs Scz Spec	.028	.101	0.29	0.776	-.169	.227
Psychopathology						
Hostility & Suspiciousness	.063	.020	3.14	0.002	.024	.102
Thinking & Perception	-.016	.012	−1.41	0.161	-.040	.007
Anxiety & Depression	.011	.008	1.24	0.216	-.006	.028
**_**constant	.103	.264	0.39	0.695	-.416	.623

*Abbreviations*: *MDD* Major Depressive Disorder, *Scz Spec* Schizophrenia Spectrum disorder, *vs* versus reference category.

Model: F (10, 184) = 3.92, p = 0.0001.

Specific QUAD areas were assessed for gender differences. Females anticipated more discrimination in housing (mean difference (MD) = 0.25, p = 0.04), education (MD = 0.35, p = 0.003), family (MD = 0.31, p = 0.03), employment (MD = 0.37, p = 0.002), and physical healthcare (MD = 0.33, p = 0.007) than males.

## Discussion

In this study, 87.6% of service users reported experienced discrimination and 92.6% anticipated discrimination in at least one life area. These figures are similar to previous research [5,7]. We also found no evidence to suggest that the overall severity of experienced discrimination differed according to diagnostic group. There have been inconsistent findings in this regard in the literature. One study using the same instrument as in our study found no difference according to diagnostic group [20]. Other studies, using different instruments to measure and operationalise experienced discrimination, have found differential rates amongst those with a diagnosis of schizophrenia or severe mental illnesses, when compared to the experiences of those with a diagnosis of depression [22,23]. These divergent findings may be due to measurement differences, but warrant replication. Interestingly, our findings are at odds with studies of public attitudes which often show more negative attitudes to individuals with a diagnosis of schizophrenia compared to depression (see [24]), and suggest that people’s actual behaviour differs from their attitudes, and/or that it is the diagnosis of a mental health problem per se, rather than specific disorders which affect levels of discrimination.

The Mixed ethnic group reported higher levels of experienced discrimination compared to the White group. While there is some evidence in the literature for differential treatment according to ethnicity in psychiatric treatment [25,26], many studies exclude Mixed ethnicities due to low sample sizes. While caution is needed in interpreting the results in the current study due to the small sample, the high rates of experienced discrimination and associations with internalised stigma suggest that further research should target these groups to explore their internal models of self and mental illness and experiences in physical and mental health care settings. Higher levels of education were associated with greater experienced discrimination. It is possible that education may lead individuals to have greater expectations or to be more critical of their experiences. Equally, higher levels of education may lead to exposure to discrimination in a greater range of life areas (e.g., through work activities) than those with less education or no qualifications.

Higher levels of experienced discrimination were associated with greater levels of anxiety and depression. As some commentators have hypothesised [12] it is possible that such symptoms may affect individuals’ interpretation of events, leading to increased reporting of unfair treatment. However, it is equally possible that experienced discrimination may lead to increased feelings of anxiety and depression. Due to the cross-sectional design it is not possible to determine the directionality of this effect; longitudinal research is therefore needed to untangle this relationship.

The anticipation of discrimination was moderately associated with experienced discrimination overall, and in most areas except employment and education. The proportion of participants reporting that employment and education areas were not applicable suggests the anticipation, and not the experience, of discrimination may lead to avoidance. This is consistent with previous research [4,8] and suggests either that negative stereotypes of employers and educators dominate people’s perceptions or that people are aware of real inequalities that people with SMI encounter (i.e., over a third of this sample for whom these areas were applicable had experienced discrimination in employment and one fifth in education). While the anticipation may be a precursor or consequence of experienced discrimination (or both) [4,8], these data suggest a need for interventions and monitoring of employment and education agencies to ensure that people with SMI are both safeguarded from unfair treatment and receive adequate support to enable them to engage in these areas. Protection against unfair treatment in employment exists in many countries (e.g., the UK Equality Act [27]) however, these data suggest low awareness of such safeguards or perhaps a distrust that employers will adhere.

Higher levels of anticipated discrimination were moderately associated with increasing age and suspiciousness, but the strongest association was with gender. Females anticipated higher levels of discrimination than males in several areas including housing, employment and family life. Previous research has linked negative future predictions with depressive cognitions (e.g., [28]) however, in this study women had higher levels of anticipated discrimination even after adjusting for depressive and anxiety symptoms (which were not independently associated with anticipated discrimination). There are a couple of possible explanations for these results. There is research to suggest that women perceive more risks than men and feel less empowered to ensure positive outcomes [29,30]. Alternatively, the anticipation of discrimination may reflect knowledge of the difficulties women with SMI report in these areas (e.g., problems finding part-time work or having children taken into care) [31,32]. These findings, in conjunction with those regarding Mixed ethnicity groups suggest that anti-stigma and discrimination interventions should be extended from public-level information campaigns, to individual-level psychological interventions assisting individuals with SMI to engage in areas they may avoid and to reduce negative appraisals of self and others where appropriate.

This study has a number of strengths. It was able to examine the association between diagnostic group and experienced discrimination more robustly than existing research. It is the first study to use a validated measure of anticipated discrimination (QUAD) enabling the examination across a broad range of areas and to address the potential influence of symptomatology, functioning and demographic variables on experienced and anticipated discrimination. There are limitations to these data. We recruited 15% of the eligible sample, however, this is typical of similar studies [7] and there were no differences between participants and eligible individuals who did not participate. In addition, we only recruited those who were engaged in secondary mental health care and therefore our study reports on those with severe illness and/or associated complex needs. In this context, our findings may not generalise to other samples that do not require regular intervention and/or are adequately treated in primary care settings. Participants knew it was a study on discrimination and therefore the sample may over-represent those with discriminatory experiences to report. Conversely, as the DISC assessed experiences in the previous 12 months, we may underestimate discrimination in areas that occur infrequently or at particular stages of life (e.g., education/parenting). Finally, the study design was cross-sectional and unable to determine the directionality of associations.

## Conclusions

Rates of experienced and anticipated discrimination amongst individuals with SMI are unacceptably high. Discrimination may lead to individuals avoiding important life areas with potential negative consequences for their overall well-being, and that of their communities. Further research is required to investigate the longitudinal associations and effects of discrimination. Interventions are needed to address structural discrimination and public stigma, but also to help service users with specific vulnerabilities and concerns regarding discrimination.

### Funding body agreements and policies

This paper presents independent research commissioned by the National Institute for Health Research (NIHR) under its Programme Grants for Applied Research scheme (RP-PG-0606-1053). The views expressed in this publication are those of the author(s) and not necessarily those of the NHS, the NIHR or the Department of Health. GT is also funded through a NIHR Specialist Mental Health Biomedical Research Centre at the Institute of Psychiatry, King’s College London and the South London and Maudsley NHS Foundation Trust.

## Competing interests

The authors declare that they have no competing interests.

## Authors’ contributions

SF, SC, CH, LH, EB, GT designed the study and wrote the protocol. JG, DJ, LD, FL collected the data. PW advised on the statistical components. SF conducted the analysis and prepared the first draft of the manuscript. All authors contributed to and approved the final manuscript.

## Pre-publication history

The pre-publication history for this paper can be accessed here:

http://www.biomedcentral.com/1471-244X/14/157/prepub
